# Hwangryun-Haedok-Tang Fermented with *Lactobacillus casei* Suppresses Ovariectomy-Induced Bone Loss

**DOI:** 10.1155/2012/325791

**Published:** 2012-10-02

**Authors:** Ki-Shuk Shim, Taesoo Kim, Hyunil Ha, Chang-Won Cho, Han Sung Kim, Dong-Hyun Seo, Jin Yeul Ma

**Affiliations:** ^1^KM-Based Herbal Drug Research Group, Korea Institute of Oriental Medicine, Daejeon 305-811, Republic of Korea; ^2^Regional Food Industry Research Group, Korea Food Research Institute, Sungnam 463-746, Republic of Korea; ^3^Department of Biomedical Engineering, Institute of Medical Engineering and Yonsei-Fraunhofer Medical Device Laboratory, Yonsei University, Wonju, Gangwon 220-710, Republic of Korea

## Abstract

Hwangryun-haedok-tang (HRT) is the common recipe in traditional Asian medicine, and microbial fermentation is used for the conventional methods for processing traditional medicine. We investigated the inhibitory effect of the *n*-butanol fraction of HRT (HRT-BU) and fHRT (fHRT-BU) on the RANKL-induced osteoclastogenesis in bone-marrow-derived macrophages. mRNA expression of osteoclastogenesis-related genes were evaluated by real-time QPCR. The activation of signaling pathways was determined by western blot analysis. The marker compounds of HRT-BU and fHRT-BU were analyzed by HPLC. The inhibitory effect of HRT or fHRT on ovariectomy-induced bone loss were evaluated using OVX rats with orally administered HRT, fHRT (300, 1000 mg/kg), or its vehicle for 12 weeks. fHRT-BU significantly inhibited RANKL-induced osteoclastogenesis, and phosphorylation of p38, IKK**α**/**β**, and NF-**κ**Bp65 compared to HRT-BU. In addition, fHRT-BU also significantly inhibited the mRNA expression of Nf**κ**b2, TNF-**α**, NFATc1, TRAP, ATPv0d2, and cathepsin K. Furthermore, administration of fHRT had a greater effect on the increase of BMD, and greater improved bone microstructure of the femora than that of HRT in ovariectomy rats. This study demonstrated that bacterial fermentation enhances the inhibitory effect of HRT on osteoclastogenesis and bone loss. These results suggest that fermented HRT might have the beneficial effects on bone disease by inhibiting osteoclastogenesis.

## 1. Introduction

Osteoclastic bone resorption and osteoblastic bone formation comprise a coupled process, known as bone remodeling [1]. It is necessary for maintenance of mineral homeostasis, bone composition, and bone microstructure in adults and the elderly. Increase of osteoclastogenesis and/or osteoclastic activity results in an increase of bone resorption over bone formation leading to an imbalance of bone remodeling, which is associated with reduction of bone mass and disruption of the bone microstructure in pathological bone diseases, including osteoporosis [2, 3]. Osteoclasts originating from the monocyte/macrophage lineage of hematopoietic stem cells differentiate into bone-resorbing multinuclear cells in the presence of the receptor activator for nuclear factor-*κ*B (NF-*κ*B) ligand (RANKL), and macrophage colony-stimulating factor (M-CSF) [4]. RANKL, a member of the TNF superfamily, is the key osteoclastogenic cytokine and M-CSF contributes to the proliferation, survival, and differentiation of osteoclast precursor cells. When RANKL interacts with its cell surface receptor, RANK, it in turn triggers the recruitment of tumor necrosis factor receptor-associated factor (TRAF) family members which serve as adapter molecules. Sequential recruitment of TRAF6 to the cytoplasmic domain of RANK leads to activation of NF-*κ*B and mitogen-activated protein (MAP) kinases, including extracellular signal-regulated kinase (ERK), c-jun-N-terminal kinase (JNK), and p38, which stimulates activated protein-1 (AP-1) activation [2, 4]. As a result of RANKL-induced activation, NF-*κ*B and AP-1 stimulate an initial expression of the key transcription factor nuclear factor of activated T cells, cytoplasmic 1 (NFATc1), which regulates gene transcription for osteoclastogenesis [5, 6]. Ectopic expression of NFATc1 causes osteoclast precursor cells to undergo efficient osteoclastogenesis in the absence of RANKL and restores osteoclastogenesis in the c-fos knockout mouse, suggesting that osteoclastogenesis signals converge on NFATc1 [7, 8].

Hwangryun-haedok-tang (HRT), also known as huang-lian-jie-du-tang or Orengedokuto, is a traditional herbal medicine prescribed for “heat clearing” from the internal organs. According to a theory in oriental medicine, HRT has been used for treating inflammatory, ulcerative disease or hypertension. The pharmacological effect of HRT is known to suppress eicosanoid biosynthesis, which results in the inhibition of inflammatory responses in carrageenan-injected air pouches model [9]. HRT also decreases intestinal injury in indomethacin-induced animal model of enteropathy through modulation of the adenosine system [10]. In addition, HRT decreases blood flow in the auricles of rats and prevents the increment of blood pressure and auricular blood flow due to theophylline [11]. However, there have been no studies done to examine the inhibitory effect of HRT on osteoclast differentiation.

Fermentation has a way to improve the therapeutic effect of traditional herbal medicine [12]. Among traditional fermentation with different sources, the beneficial effect of bacterial or fungus fermentation on medicinal herb has been suggested. *Anoectochilus formosanus* Hayata fermented with *Lactobacillus acidophilus* causes an enhancement of the antioxidant effect accompanied with an increase in total phenols [13]. In addition, anabolic activity of antlers on osteoblast differentiation is enhanced by fermentation with *Cordyceps militaris*, which increases the production of the active component, a sialic acid [14]. Interestingly, bacterial fermentation of carbohydrates or milk could have a positive effect on bone mineral content through increase of mineral solubility, expression of calcium-binding protein, and generation of bone-modulating factors, which prevents bone loss in animal models [15, 16]. Based on these studies, it could be suggested that fermentation stimulates the production or conversion of the primary active components to its metabolite in medicinal herb, which may increase the therapeutic effect of herbal medicine. However, no research has been conducted to study whether bacterial fermentation could improve the pharmacological effect of traditional herbal medicine on bone disease.

In a preliminary experiment, we found that the fermented HRT by lactobacteria (fHRT) has significantly more inhibitory effect on osteoclastogenesis than non-fermented HRT (HRT). Furthermore, we selected *n*-butanol as the organic solvent for maximizing the inhibitory effect of fHRT on osteoclastogenesis. In this study, we investigated the inhibitory effect and the molecular mechanism of *n*-butanol extract of fHRT (fHRT-BU) on RANKL-induced osteoclastogenesis in mouse bone-marrow-derived macrophages (BMMs). Ovariectomy (OVX) rat is a typical animal model of bone loss which has similar characteristics of postmenopausal osteoporosis: reduction in bone mineral density (BMD) and an increased rate of bone turnover with osteoclast bone resorption exceeding formation [17, 18]. To evaluate the therapeutic potential of fHRT for treating bone loss, we examined the inhibitory effect of HRT and fHRT in OVX-induced bone loss.

## 2. Materials and Methods

### 2.1. Chemicals and Reagents

*Coptis japonica* Makino, *Scutellaria baicalensis* Georgi, *Phellodendron chinense* Schneider, and *Gardenia jasminoides fructus* were purchased from the Korea Medicine Herbs Association (Yeongcheon, Gyeongsangbuk-do, Republic of Korea). General aerobic medium (de Man, Rogosa and Sharpe broth: MRS) and MRS agar were purchased from Difco Co. (Detroit, MI, USA). *α*-modified minimal essential medium (*α*-MEM), fetal bovine serum (FBS), antibiotics (100 U/mL penicillin and 100 *μ*g/mL streptomycin), BCA protein assay kit, and SuperSignal West Femto Maximum Sensitivity Substrate were purchased from Thermo Fisher Scientific Inc. (Rockford, IL, USA). A Cell Counting Kit-8 (CCK-8) was obtained from Dojindo Molecular Technologies (Rockville, MD, USA). RNA-spin total RNA extraction kit was purchased from Intron (Daejeon, Republic of Korea). AccuPower RT PreMix and AccuPower GreenStar qPCR Master Mix were obtained from Bioneer (Daejeon, Republic of Korea). Baicalin, berberine, 1,25(OH)_2_D_3_, palmatine, polybrene, puromycin, *q*-nitrophenyl phosphate, and trifluoroacetic acid (TFA) were purchased from Sigma Chemical Co. (St. Louis, MO, USA). Antibodies specific for phospho-ERK1/2 (Thr202/Tyr204), ERK, phospho-JNK1/2 (Thr183/Tyr185), JNK, phospho-p38 (Thr180/Tyr182), p38, phospho-IKK*α*/*β*, I-*κ*B*α*, phospho-p65 (Ser536), and p65 were purchased from Cell Signaling Technology (Danvers, MA, USA). Antibodies against NFATc1 (7A6) and c-fos (H-125) were purchased from Santa Cruz Biotechnology (CA, USA). A PVDF membrane was purchased from Millipore (Darmstadt, Germany). Geniposide was purchased from Wako Pure Chemical Industries, Ltd. (Japan). HPLC grade water and methanol were purchased from J. T. Baker (Phillipsburg, NJ, USA).

### 2.2. Animals

Specific pathogen-free (SPF) Sprague Dawley female rats, 10 weeks of age, were purchased from Samtako Bio Inc. (Seoul, Republic of Korea). All rats were housed at 22 ± 1°C and 55 ± 10% humidity on a 12-h light/dark cycle with free access to food and water. Animal experiments were carried out in accordance with the National Institute of Health's Guidelines for the Care and Use of Laboratory Animals. The experiments were approved by the Institutional Animal Care and Use Committee at the Korea Institute of Oriental Medicine.

### 2.3. Preparation of HRT

All four constituents of HRT, *Coptis japonica* Makino 250 g, *Scutellaria baicalensis* Georgi 250 g, *Phellodendron chinense* Schneider 250 g, and *Gardenia jasminoides fructus* 250 g were used in this study. All voucher specimens were deposited in the herbal bank of the Center for Herbal Medicine Improvement Research, Korea Institute of Oriental Medicine. HRT was prepared by using a water extraction method (Gyeongseo Extractor Cosmos-600, Inchon, Republic of Korea). The total quantity of four herbs was placed in 10 L of distilled water for 1 h, and then extracted by heating for 3 h at 115°C. After extraction, HRT was filtered out using standard testing sieves (106 *μ*m) (Retsch, Haan, Germany) and stored at 4°C before use.

### 2.4. Fermentation and Fractionation of HRT

*Lactobacillus casei* KFRI-127 used in this study was derived from Korea Food Research Institute (KFRI, Seongnam-si, Republic of Korea). After two successive transfers of the test organisms in MRS broth at 37°C for 24 h, the activated cultures were again inoculated into broth. It was then properly diluted to obtain an initial population of 1–5 × 10^6^ CFU/mL and served as the inoculum. Viable cell counts of the strains were determined in duplicate by using the pour-plate method on MRS agar. For fermentation, 5 mL of HRT in a test tube with cap was inoculated with 0.05 mL of the inoculums as described above. This was incubated at 37°C for a period of 48 h. HRT or fHRT was fractionated by successive solvent extraction with *n*-hexane, ethylacetate, and *n*-butanol. Each fraction was then evaporated to dryness under vacuo and stored in desiccators at −20°C before use. The *n*-butanol-soluble fraction of HRT and fHRT was designated as HRT-BU (yield about 16.45%) and fHRT-BU (yield about 17.72%), respectively.

### 2.5. Cell Culture and Cell Viability Assay

Murine osteoclasts were prepared from bone marrow cells as described previously [2]. Bone marrow cells (BMCs) were cultured in *α*-MEM containing 10% FBS with M-CSF (60 ng/mL) and antibiotics (100 U/mL penicillin and 100 *μ*g/mL streptomycin) for 3 days, and the attached BMMs were used as osteoclast precursors. To evaluate cell viability, cells (1 × 10^4^ cells/well) were incubated with different concentrations of sample (12.5–200 *μ*g/mL) in the presence of M-CSF (60 ng/mL) for 3 days on 96-well plates. A CCK-8 was used to examine cell viability according to the manufacturer's protocol (Dojindo Molecular Technologies). Data represented the mean ± SD of triplicate.

### 2.6. TRAP Assay and Osteoclast Formation

To generate osteoclasts, BMMs were cultured with M-CSF (60 ng/mL) and RANKL (150 ng/mL) for 4 days. Total TRAP activity was measured at an absorbance of 405 nm after treatment with substrate (*p*-nitrophenyl phosphate) as described previously [19]. TRAP-positive multinucleated cells (TRAP(+)MNCs) containing more than three nuclei and larger than 100 *μ*m in diameter were counted.

### 2.7. Real-Time Quantitative PCR (QPCR)

To evaluate NF-*κ*B-regulated gene expression, cells (3 × 10^5^ cells/well in a 6-well plate) were preincubated with sample for 2 h in the presence of M-CSF (60 ng/mL) and then stimulated with RANKL (150 ng/mL) for indicated times. To evaluate NFATc1-regulated gene expression, cells (3 × 10^5^ cells/well in a 6-well plate) were coincubated with sample for indicated times in the presence of M-CSF and RANKL. Total RNA was isolated with an RNA-spin total RNA extraction kit according to the manufacturer's protocol. First-strand cDNA was synthesized from 1 *μ*g of total RNA in AccuPower RT PreMix according to the manufacturer's protocol. Subsequently, SYBR green-based QPCR amplification was performed using cDNA diluted to 1 : 3, 10 pmol of primers, and AccuPower GreenStar qPCR Master Mix in the Applied Biosystems 7500 Real-Time PCR System (Applied Biosystems, Foster City, CA, USA) according to the manufacturer's protocol. The PCR reaction consisted of three segments. In the first segment, the polymerase was activated by heating at 95°C for 10 min. The second segment consisted of 40 cycles of 94°C for 30 s, and 60°C for 1 min. The third segment, designed to generate PCR product-temperature dissociation curves (melting curves), consisted of 95°C for 1 min, 60°C for 30 s, and 95°C for 30 s. All reactions were run in triplicate, and data were analyzed using the 2^−ΔΔCT^ method. HPRT was used as the internal standard.

### 2.8. Western Blot Analysis

BMMs were incubated with sample for indicated time, washed with ice-cold phosphate-buffered saline (PBS), and lysed in protein extraction buffer consisting of 50 mM Tris-HCl (pH 8.0), 5 mM EDTA, 150 mM NaCl, 1% NP-40, 0.1% SDS, 1 mM PMSF, one protease-inhibitor cocktail tablet, and one phosphatase-inhibitor cocktail tablet. Cell lysates were centrifuged at 10 000 ×g for 15 min at 4°C. Protein concentration was determined with a BCA protein assay kit. Protein samples were mixed with sample buffer (100 mM Tris-HCl (pH 7.6), 2% SDS, 1% 2-mercaptoethanol, 2% glycerol, and 0.01% bromophenol blue), incubated at 95°C for 5 min, and loaded onto 10% polyacrylamide gels. Electrophoresis was performed using a Mini-PROTEAN 3 Cell (Bio-Rad, Hercules, CA, USA). Resolved proteins were transferred onto a PVDF membrane. The membrane was first incubated in blocking buffer (10 mM Tris-HCl (pH 7.5), 150 mM NaCl, 0.1% Tween 20, and 5% non-fat dry milk) and then incubated for 2 h at room temperature with 1000 diluted primary antibodies. After washing with a washing buffer (10 mM Tris-HCl (pH 7.5), 150 mM NaCl, and 0.1% Tween 20) three times for 10 min each, the membrane was probed with 2000 diluted secondary antibody for 1 h. The membrane was then washed with a washing buffer three times for 10 min each and developed with SuperSignal West Femto Maximum Sensitivity Substrate. Chemiluminescent signals were detected with a LAS-4000 luminescent image analyzer (Fuji Photo Film Co., Japan).

### 2.9. High-Performance Liquid Chromatography (HPLC) Analysis

The high-performance liquid chromatography (HPLC) DAD system (Hitachi Co., Japan) consisted of a pump (L-2130), autosampler (L-2200), column oven (L-2350), and diode array UV/VIS detector (L-2455). The output signal of the detector was recorded using an EZChrom Elite software by Hitachi. Chromatographic separation was achieved in a Luna C18 column (4.6 mm × 250 mm, 5 *μ*m, Phenomenex Co., USA) at 254 and 380 nm. The mobile phase was 100% methanol (A) and water-containing 0.1% trifluoroacetic acid (B) with a step gradient elution (0–5 min, 5% A; 5–70 min, 100% A, 70–80 min, 100% A). The column temperature was maintained at 40°C. Each standard solution was prepared by dissolving each marker components, geniposide, berberine, palmatine, and baicalin, with 100% methanol at the concentration of 100 *μ*g/mL. Prior to analysis, the sample preparation was dissolved with 100% methanol at the concentration of 10 mg/mL and filtered through a 0.45 *μ*m filter. 3 *μ*L of samples was injected for the HPLC analysis.

### 2.10. Animal Model of Ovariectomized Rats

Female rats were either sham operated (sham, *n* = 8) or surgically OVX (OVX, *n* = 40) after acclimatization in the laboratory environment for one week. One week after OVX surgery, the OVX rats were randomly divided into five groups with eight rats each: (1) OVX: bilaterally OVX; (2) HRT-0.3: bilaterally OVX followed by 0.3 g/kg of HRT administration; (3) HRT-1.0: bilaterally OVX followed by 1.0 g/kg of HRT administration; (4) fHRT-0.3: bilaterally OVX followed by 0.3 g/kg of fHRT administration; and (5) fHRT-1.0: bilaterally OVX followed by 1.0 g/kg of fHRT administration. The administration of samples began 1 week and finished 3 months after OVX surgery. The same amount of saline was orally administered to the sham and OVX groups. Animal experiments were carried out in accordance with the National Institute of Health's Guidelines for the Care and Use of Laboratory Animals. The experiments were approved by the Institutional Animal Care and Use Committee at the Korea Institute of Oriental Medicine.

### 2.11. Microcomputed Tomography (Micro-CT) Analysis

To determine 3-dimensional bone structure *in vivo*, we performed histomorphometric analysis. Computed tomographic images of the femur of each rat were acquired 3 months after OVX surgery, using the *In-Vivo* Micro-CT (SkyScan 1076, SkyScan N.V., Belgium) at a resolution of 18 *μ*m, with the following parameters: 100 kV, 100 mA, 1,770 ms. The beam-hardening errors were corrected to improve the quality of the micro-CT images by flat-field correction before scanning and beam-hardening correction during reconstruction. Three-dimensional models of the trabecular bones of the femur were reconstructed using SkyScan CT Analyzer version 1.11 to evaluate the alteration of bone. In addition, the structural parameters were measured on the trabecular bone of the femora.

### 2.12. Statistical Analysis

SPSS software (SPSS Inc., Chicago, IL, USA) was used for all statistical analyses. The significance of difference in TRAP activity and the mRNA expression levels of osteoclast differentiation-related genes were determined using Student's *t*-test using HPRT-normalized 2^−ΔΔCT^ values. For the statistical analysis of the results of the animal experiment, a parametric one-way analysis of variance was used to test for any differences among the groups. Duncan's multiple comparison test was used to confirm significant differences in the mean value between the groups. A *P*-value less than 0.05 was considered significant.

## 3. Results

### 3.1. Effect of HRT and fHRT on Osteoclastogenesis in BMMs

**Figure 1:**
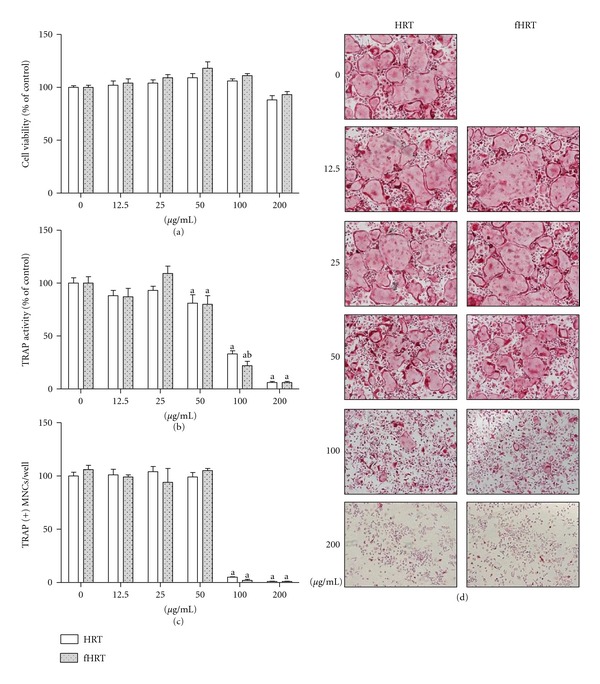
HRT and fHRT inhibit RANKL-induced osteoclast formation in BMMs. BMMs (1 × 10^4^ cells/well in a 96-well plate) were incubated with M-CSF (60 ng/mL), RANKL (150 ng/mL), and the indicated concentrations of HRT or fHRT for 4 days. Cell viability was measured by CCK-8 assay (a). The cells were fixed and stained for TRAP activity (b), and then the number of TRAP-positive multinuclear osteoclast cells (TRAP(+)MNCs) (c) with more than 3 nuclei and larger than 100 *μ*m in diameter were counted. Representative microscopic pictures of multinucleated osteoclasts (d) were shown at a magnification of 100x. Data represents mean ± SD of three independent experiments. ^a^ *P* < 0.05, versus vehicle treated control. ^b^ *P* < 0.05, versus HRT.

**Figure 2:**
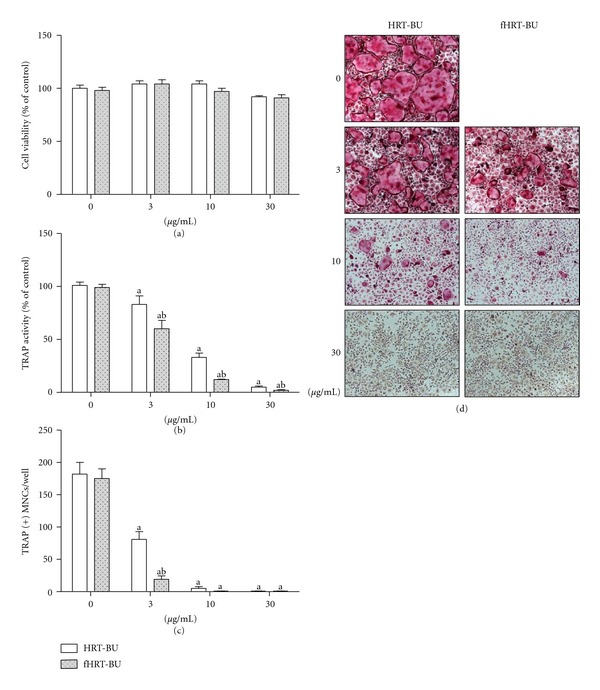
HRT-BU and fHRT-BU inhibit RANKL-induced osteoclast formation in BMMs. BMMs (1 × 10^4^ cells/well in a 96-well plate) were incubated with M-CSF (60 ng/mL), RANKL (150 ng/mL), and the indicated concentrations of HRT-BU or fHRT-BU for 4 days. Cell viability was measured by CCK-8 assay (a). The cells were fixed and stained for TRAP activity (b) and then the number of TRAP-positive multinuclear osteoclast cells (TRAP(+)MNCs) (c) with more than 3 nuclei and larger than 100 *μ*m in diameter were counted. Representative microscopic pictures of multinucleated osteoclasts (d) were shown at a magnification of 100x. Data represents mean ± SD of three independent experiments. ^a^ *P* < 0.05, versus vehicle treated control. ^b^ *P* < 0.05, versus HRT.

We first evaluated the effect of HRT or fHRT on the cell viability of BMMs to examine any potential cytotoxic effect. This result showed that HRT or fHRT had no cytotoxic effect on mouse BMMs in a wide range of concentrations (12.5–200 *μ*g/mL) (Figure 1(a)). To evaluate the inhibitory effect of HRT or fHRT on RANKL-induced osteoclast differentiation, we examined TRAP activity and the number of TRAP-positive multinuclear cells. HRT or fHRT significantly decreased TRAP activity in a dose-dependent manner (Figure 1(b)). In particular, 100 *μ*g/mL concentration of fHRT had a greater inhibitory effect on TRAP activity (15%) than that of HRT (Figures 1(c) and 1(d)). Furthermore, to maximize the inhibitory effect of fHRT on osteoclastogenesis, we made extracts by fractionating components according to their polarity and compared their effect on osteoclastogenesis. *n*-butanol extracts of fHRT proved to be highly effective in reducing formation of TRAP-positive multinuclear osteoclasts while the other extracts had lower activity or cytotoxicity (data not shown). After fractionation with *n*-butanol, the inhibitory effect of fHRT increased more than 10-fold. We next compared the inhibitory effect of HRT-BU and fHRT-BU on osteoclastogenesis in mouse BMMs. HRT-BU (3–30 *μ*g/mL) significantly decreased RANKL-induced TRAP activity without cytotoxicity in a dose-dependent manner (Figures 2(a) and 2(b)). Interestingly, fHRT-BU at all concentration had about 20% greater inhibitory effects on TRAP activity than HRT-BU. In addition, 3 *μ*g/mL concentration of HRT-BU markedly suppressed about 70% of TRAP-positive multinuclear osteoclast formation induced by RANKL (Figures 2(c) and 2(d)). fHRT-BU completely inhibited multinuclear osteoclast formation at 3 *μ*g/mL. We subsequently used 10 *μ*g/mL of HRT-BU and fHRT-BU in the following study.

### 3.2. Effect of HRT-BU and fHRT-BU on NFATc1 and c-fos Expression

**Figure 3:**
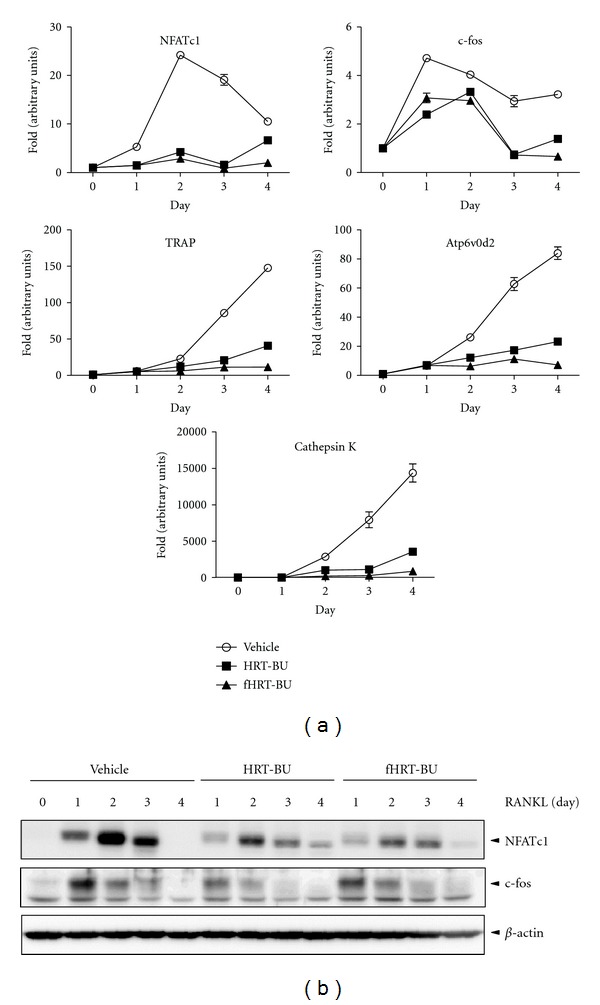
fHRT-BU inhibits RANKL-induced NFATc1 expression in BMM cells. (a) BMMs (3 × 10^5^ cells/well in a 6-well plate) were incubated with HRT-BU (10 *μ*g/mL) and RANKL (150 ng/mL) or fHRT-BU and RANKL for the indicated times. Total RNA was isolated on the indicated time and mRNA expression of NFATc1, c-fos, TRAP, ATPv0d2, and cathepsin K was analyzed by QPCR. (b) Whole cell lysates (30 *μ*g) were analyzed by western blot analysis with antibody specific for NFATc1 and c-fos. *β*-actin was used as loading control. Data are representative of three independent experiments.

NFATc1 and c-fos are essential transcription factors for osteoclast differentiation. To determine the inhibitory mechanism of fHRT-BU on osteoclast differentiation, we explored whether fHRT-BU inhibits RANKL-induced NFATc1 and c-fos expression. As compared to the vehicle, HRT-BU and fHRT-BU significantly decreased RANKL-induced mRNA level of NFATc1 and c-fos (Figure 3(a)). We also analyzed mRNA expression of osteoclastogenesis-related genes (TRAP, ATPv0d2, and cathepsin K) regulated by NFATc1. HRT-BU and fHRT-BU significantly decreased RANKL-induced mRNA level of these genes. Specifically, fHRT-BU had a greater inhibitory effect on mRNA level of TRAP, ATPv0d2, and cathepsin K compared with HRT-BU (Figure 3(a)). To confirm these inhibitory effects of HRT-BU and fHRT-BU on NFATc1 and c-fos expression, the protein expression of NFATc1 and c-fos was determined by western blot analysis. HRT-BU and fHRT-BU inhibited RANKL-induced NFATc1 protein expression, but not c-fos (Figure 3(b)).

### 3.3. Effect of HRT-BU and fHRT-BU on RANKL-Induced MAP Kinases and NF-*κ*B Signaling

**Figure 4:**
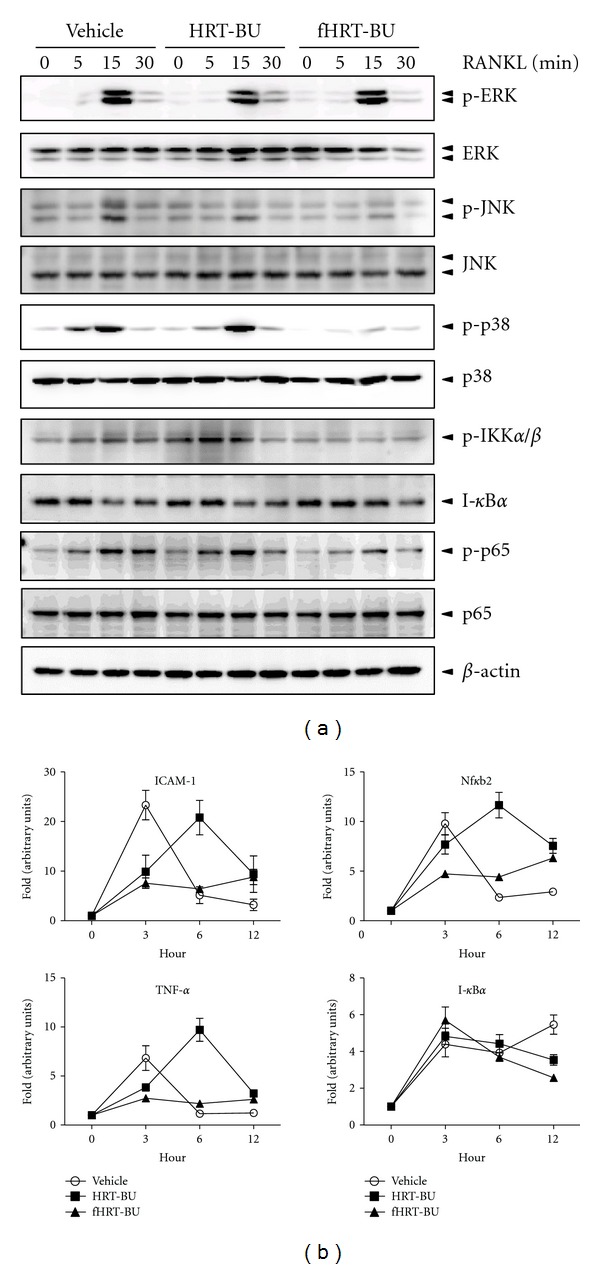
fHRT-BU inhibits RANKL-induced p38, IKK*α*/*β*, and p65 phosphorylation in BMM cells. (a) BMMs (3 × 10^5^ cells/well in a 6-well plate) were pretreated with HRT-BU or fHRT-BU (10 *μ*g/mL) for 2 h and then stimulated with RANKL (150 ng/mL) for the indicated times. Whole cell lysates (10 *μ*g) were analyzed by western blot analysis with indicated antibodies. MAP kinases, IKK*α*/*β*, and p65 activation was represented by the levels of protein phosphorylation. *β*-actin was used as loading control. (b) BMMs were pretreated with HRT-BU or fHRT-BU (10 *μ*g/mL) for 2 h and then stimulated with RANKL (150 ng/mL) for 3, 6, and 12 h. Total RNA was isolated on the indicated time and mRNA expression of ICAM-1, TNF-*α*, Nf*κ*b2, and I-*κ*B*α* was analyzed by QPCR. Data represents mean ± SD of three independent experiments.

MAP kinases (ERK, JNK, and p38), IKK*α*/*β*, I-*κ*B*α*, and p65 (Ser-536) mediating the signaling pathways play an important role in RANKL-induced osteoclastogenesis. To examine whether HRT-BU and fHRT-BU affect immediate early signaling pathways, BMMs were treated with HRT-BU or fHRT-BU and then were stimulated with RANKL for the indicated periods of time. Both HRT-BU and fHRT-BU decreased the RANKL-induced activation of JNK. Furthermore, fHRT-BU markedly suppressed p38, IKK*α*/*β*, and p65 phosphorylation compared with RANKL and HRT-BU treatment (Figure 4(a)).

p38 participates in NF-*κ*B transactivation by mediating p65 phosphorylation [20]. Since fHRT-BU inhibited p38 and p65 (Ser-536) phosphorylation, we explored whether fHRT-BU inhibits NF-*κ*B-dependent transcription in BMMs. QPCR was applied to measure mRNA expression level of four genes (ICAM-1, Nf*κ*b2, TNF-*α*, and I-*κ*B*α*) regulated by NF-*κ*B. As compared to HRT-BU, fHRT-BU had a greater inhibitory effect on RANKL-induced mRNA level of Nf*κ*b2, and TNF-*α* of about 1.5-fold at 3 h after RANKL treatment (Figure 4(b)).

### 3.4. HPLC Analysis of HRT-BU and fHRT-BU

**Figure 5:**
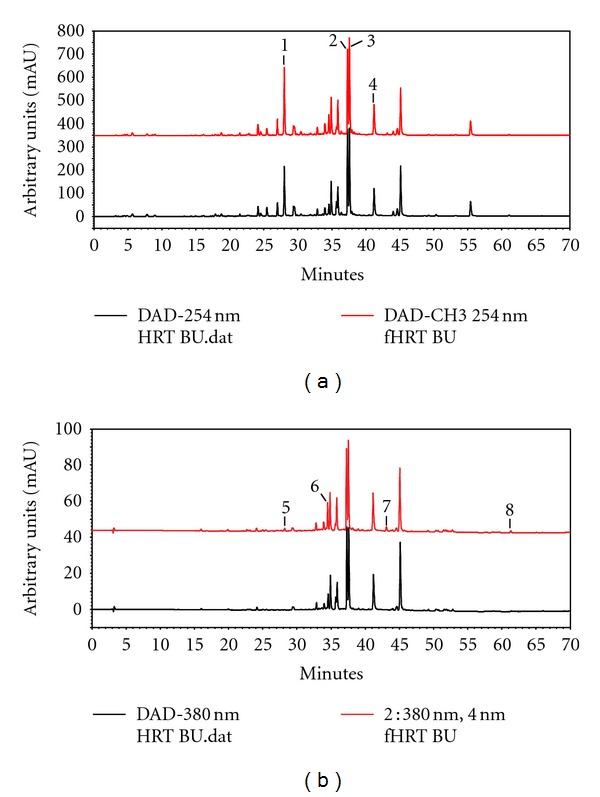
The HPLC analysis chromatograms of HRT-BU and fHRT-BU at 254 nm (a) and 380 nm (b). (1) Geniposide *t*_*R*_ 27.96 min; (2) berberine *t*_*R*_ 37.27 min; (3) palmatine *t*_*R*_ 37.54 min; (4) baicalin *t*_*R*_ 41.16 min; (5) *t*_*R*_ 28.18 min; (6) *t*_*R*_ 34.50 min; (7) *t*_*R*_ 43.08 min; (8) *t*_*R*_ 61.30 min.

HRT-BU and fHRT-BU significantly inhibited RANKL-induced osteoclastogenesis and NFATc1 expression in BMMs. Flavonoids have been suggested as potential components related to the therapeutic effect of the medicinal herbs. Therefore, we performed HPLC analysis to characterize HRT-BU and fHRT-BU by fingerprinting the marker components of medicinal herbs in HRT. HPLC analysis successfully separated the major peaks and the change of peaks in HRT-BU and fHRT-BU (Figure 5). HPLC analysis chromatograms simultaneously identified major components of both samples that were geniposide (1, *t*_*R*_ 27.96 min), berberine (2, *t*_*R*_ 37.27 min), palmatine (3, *t*_*R*_ 37.54 min), and baicalin (4, *t*_*R*_ 41.16 min). There was no significant change of major components between HRT-BU and fHRT-BU. However, the intensity of four unidentified compounds, the peak numbers 5 to 8, was increased in fHRT-BU compared to HRT-BU.

### 3.5. The Quantification of Bone Mass by Micro-CT in fHRT-Treated OVX Rats

**Figure 6:**
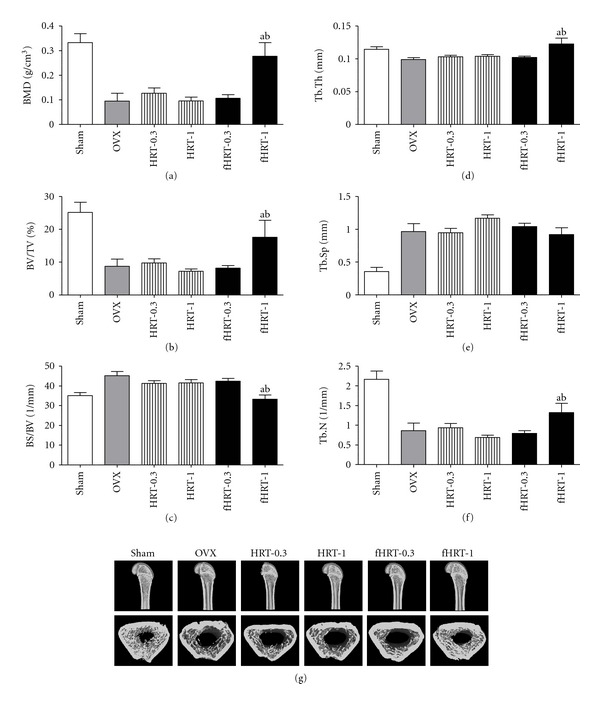
fHRT increases bone density in OVX rats. Bone mineral density (BMD) and structural parameter of trabecular bone of femur were analyzed by micro-CT after 12 weeks of HRT and fHRT administration. Graphs represented BMD (a), bone volume (BV/TV, (b)), bone surface (BS/BV, (c)), trabecular thickness (Tb.Th, (d)), trabecular separation (Tb.Sp, (e)), and trabecular number (Tb.N, (f)). Representative micro-CT images of distal metaphysic femur of sham, OVX, HRT-0.3, HRT-1.0, fHRT-0.3, and fHRT-1.0 group (g). ^a^ *P* < 0.05, versus OVX. ^b^ *P* < 0.05, versus HRT-1.0.

To examine the potential inhibitory effect of HRT and fHRT on OVX-induced bone loss, bone mass of femur was analyzed by micro-CT. In comparison with the sham group, the OVX group exhibited significant decrease in BMD of about 70% (Figure 6(a)). Total BMD in fHRT-1.0 groups was significantly higher than that of the OVX and HRT-1.0 group. Particularly, fHRT-1.0 groups showed a 55% and 54% greater BMD than the OVX and HRT-1.0 groups, respectively. The structural parameters of trabecular bone for bone volume/trabecular volume (BV/TV), bone surface/bone volume (BS/BV), trabecular thickness (Tb.Th), trabecular separation (Tb.Sp), and trabecular number (Tb.N) were shown in Figure 6. Compared to the sham group, the OVX group showed significant change in BV/TV (−65%), BS/BV (+29%), Tb.Th (−14%), Tb.Sp (+169%), and Tb.N (−60%). There was no significant change of these parameters in the HRT-0.3 and HRT-1.0 groups. Interestingly, the fHRT-1.0 group showed significant improvement of the decreasing structural parameters induced by OVX. Particularly, fHRT-1.0 group showed significant improvement of all structural parameters over the HRT-1.0 group, recording a 41% greater BV/TV, 23% less BS/BV, 15% greater Tb.Th, but no significant differences in Tb.Sp and Tb.N. Furthermore, the femur of the fHRT-1.0 group formed a tight and dense structure compared with that of the OVX and HRT-1.0 group (Figure 6(g)).

## 4. Discussion

The interaction of RANKL with its receptor, RANK, initiates early signaling pathways including MAP kinases and the NF-*κ*B pathway through the adaptor protein TRAF in osteoclastogenesis. In this study, we found that fHRT markedly decreased the RANKL-induced phosphorylation of p38, IKK*α*/*β*, and NF-*κ*B p65 (Ser-536) (Figure 4(a)). NF-*κ*B p65 and p50 are recruited to the NFATc1 promoter with NFATc2 for autoamplification of NFATc1 expression in RANKL-induced osteoclast differentiation [21]. IKK*α*/*β* and p38 are both necessary for enhanced NF-*κ*B p65 transactivation, whereas mutation of either Ser-529 or Ser-536 of p65 abolishes this effect [22]. In particular, p38 participates in the RANKL-stimulated NFATc1 induction, which is mediated by NF-*κ*B p65 activation on Ser-536 [20]. NFATc1 is a master transcription factor regulating the expression of osteoclastogenesis-related gene in osteoclasts [4, 8]. Because fHRT significantly decreased RANKL-induced p38 and NF-*κ*B p65 (Ser-536) activation, and mRNA expression of NF-*κ*B regulated genes (Figure 4), fHRT might inhibit NF-*κ*B activity in osteoclast precursor cells, resulting in the inhibition of osteoclastogenesis.

Consistent with the inhibitory effect of fHRT on MAP kinase (JNK and p38) and NF-*κ*B (IKK*α*/*β* and NF-*κ*B p65) pathway (Figure 4), we also found that fHRT-BU significantly augmented the suppressive effect of HRT-BU on NFATc1 expression, both in mRNA and on the protein level (Figure 3). In addition, fHRT significantly inhibited NFATc1-regulated and osteoclast-specific genes (Figure 3(a)) including TRAP, Atp6v0d2, and cathepsin K that modulate osteoclast fusion, activation, and function [23–25]. ERK activates c-fos, while JNK increases AP-1 transcriptional activity through the phosphorylation of c-Jun [26]. p38 induces NF-*κ*B transactivation for NFATc1 expression [20]. In response to RANKL and TNF, c-fos which is downstream of NF-*κ*B transcriptionally regulates NFATc1 expression [6]. Therefore, as fHRT significantly suppressed mRNA and protein expression of NFATc1, fHRT might target NFATc1 expression and NFATc1-dependent gene expression through inhibition of p38 and NF-*κ*B p65 signaling in RANKL-induced osteoclastogenesis.

Bacterial fermentation results in not only the structure change of flavonoids, stimulating its bioavailability and metabolism [27], but also the generation of components having bone-preserving potential. In this study, we found that the bacterial fermentation changes the contents of flavonoid in fHRT-BU (Figure 5) and increases the inhibitory effect of HRT on bone loss (Figure 6). Our finding is consistent with those of previous studies and indicates that bacterial fermentation produces the inhibitory molecules on bone loss. 1,4-dihydroxy-2-naphthoic acid or 6-*O*-succinylated isoflavone glycoside generated by bacterial fermentation improves bone mass reduction through inhibition of bone resorption and/or bone-resorption-related cytokine levels [28, 29]. The flavonoids undergo a cleavage of the glycosides by bacterial metabolism in the small intestine, which is followed by absorption and metabolism of the aglycone [30]; they may contribute to the beneficial effect on bone health [16]. Thus, it could be suggested that lactic fermentation might increase the generation and bioavailability of the inhibitory components in HRT on osteoclastogenesis, which consequently contributes to enhanced characteristics of bone structure and an increase of BMD in OVX rats.

The present study evaluated whether the bacterial fermentation affects the inhibitory effect of HRT on OVX-induced bone loss. We found that lactic fermented HRT, but not HRT, significantly inhibited the OVX-induced decrease of BMD, BV/TV, Tb.Th, and Tb.N (Figure 6). In general, a significant loss of BV/TV is associated with a loss of Tb.N and Tb.Th in OVX rats, indicating the loss of a finer network of the numerous trabeculae in the metaphyseal region, and the increase of osteoclastic bone resorption [31]. Estrogen maintains trabecular bone volume by suppression of bone resorption and stimulation of bone formation, which consequently suppresses OVX-induced increase of bone turnover [32, 33]. Traditional herbs are known to have some flavonoid with phytoestrogenic activity that is structurally similar to estrogen and interacts with estrogen receptor. Phytoestrogen directly reduces osteoclast activity but stimulates osteoblastogenesis, and dose-dependently decreases bone turnover in the OVX model [34]. In addition, phytoestrogen reduces the postmenopausal characteristics associated with an increase in obesity and vaginal atrophy due to estrogen deficiency. However, fHRT did not reverse estrogen-deficiency-induced obesity and vaginal atrophy (data not shown). Thus, it would be possible that bioactive compounds in fHRT might have positive estrogenic effects on bone without a uterotrophic effect as reported in previous studies [28, 35].

In conclusion, we first found that lactic bacterial fermentation of HRT inhibits OVX-induced bone loss by enhancing bone mineral density and bone microstructure. It might result from the inhibitory effect of fHRT on osteoclastogenesis by downregulating NFATc1 expression. The results of this study suggest that bacteria-fermented HRT could have therapeutic potential for the treatment of postmenopausal osteoporosis and other bone diseases. Further studies would have to be carried out in order to determine the active components in fHRT.
